# Combination of Oral Doxycycline and Rifampin for Pan‐Drug Resistant *Klebsiella pneumoniae* Infection: A Case Report

**DOI:** 10.1002/ccr3.73238

**Published:** 2026-07-25

**Authors:** Ali Akbar Heydari, Sepideh Elyasi, Farshad Abedi

**Affiliations:** ^1^ Research Center for Infection Control & Hand Hygiene, Department of Infectious Diseases, Imam Reza Hospital Mashhad University of Medical Sciences Mashhad Iran; ^2^ Department of Clinical Pharmacy, School of Pharmacy Mashhad University of Medical Sciences Mashhad Iran

**Keywords:** bacterial drug resistance, doxycycline, *Klebsiella pneumoniae*, osteomyelitis, rifampin, wound infection

## Abstract

Pan‐drug resistant *Klebsiella pneumoniae* is a major therapeutic challenge, particularly when standard last‐line agents are unavailable or contraindicated. We report a 45‐year‐old man with multifocal osteomyelitis and persistent purulent wound discharge despite broad‐spectrum antibiotics, repeated debridement, and two sequential amputations. Wound culture grew *K. pneumoniae* resistant to all antibiotics tested in the locally available susceptibility panel. Initial treatment with colistin plus extended‐infusion meropenem was discontinued because of acute kidney injury. Oral doxycycline 100 mg twice daily combined with rifampin 600 mg once daily was then initiated as salvage therapy, resulting in rapid resolution of purulent discharge and marked reduction in inflammatory markers. This case suggests that oral doxycycline plus rifampin may represent a low‐cost and accessible salvage option for highly resistant *K. pneumoniae* infections when standard therapies are unavailable or not tolerated.

## Key Clinical Message

Combination therapy with oral doxycycline and rifampin may represent a potential salvage option for managing pan‐drug‐resistant *Klebsiella pneumoniae* wound infections when standard last‐line antibiotics are unavailable or contraindicated. Our case demonstrates a rapid clinical and biochemical response to this inexpensive and accessible therapeutic regimen.

## 1 Introduction

The increasing number of multidrug resistant (MDR) and pan‐drug resistant (PDR) Gram‐negative bacteria with the decline in effective treatments is a global health issue. *Escherichia coli* , *Pseudomonas aeruginosa* , *Acinetobacter baumannii* , and *Klebsiella pneumoniae* are among the highly resistant Gram‐negative pathogens usually identified in hospitalized patients [1]. Several combinations with newer beta‐lactam/beta‐lactamase inhibitors, polymyxins, tetracyclines, and aminoglycosides have now been proposed by the Centers for Infectious Disease Control and Prevention [2]. However, the combination of last‐line antibiotics often fails to eradicate pathogens and nearly always occurs concomitantly with serious adverse effects such as nephrotoxicity. So, morbidity and mortality are still high in patients infected with resistant Gram‐negative bacteria, demonstrating the need for investigating and developing newer approaches.

## 2 Case History/Examination

We report a case of a 45‐year‐old man with chills and fever, as well as multiple wounds with purulent discharge mainly in the posterolateral of right knee, who was admitted to the Infectious Ward of Imam Reza Hospital, Mashhad, Iran in October 2023. He was homeless and an intravenous drug user. In past medical history, he had a leg fracture in the right femoral bone following an accident and hepatitis C virus (HCV) antibody was positive.

## 3 Differential Diagnosis, Investigations and Treatments

Screening for other causes of immunosuppression, including HIV testing and other immune markers, was performed and all results were negative. Laboratory investigations revealed white blood cell (WBC) 19.5 × 109/L (neutrophils 95%; lymphocytes 3.7%), hemoglobin 9.2 g/dL, platelets 227 × 109/L, urea 59 mg/dL, creatinine 1.1 mg/dL, sodium 126 mEq/L, potassium 3.3 mEq/L, random blood glucose 72 mg/dL, aspartate aminotransferase (AST) 14 U/L, alanine transaminase (ALT) 7 U/L, alkaline phosphatase (ALP) 101 U/L, serum albumin 3 g/dL, erythrocyte sedimentation rate (ESR) 143 mm/h, C‐reactive protein (CRP) 280 mg/L (normal range, 0–8 mg/L), and lactate dehydrogenase (LDH) 520 U/L.

**FIGURE 1:**
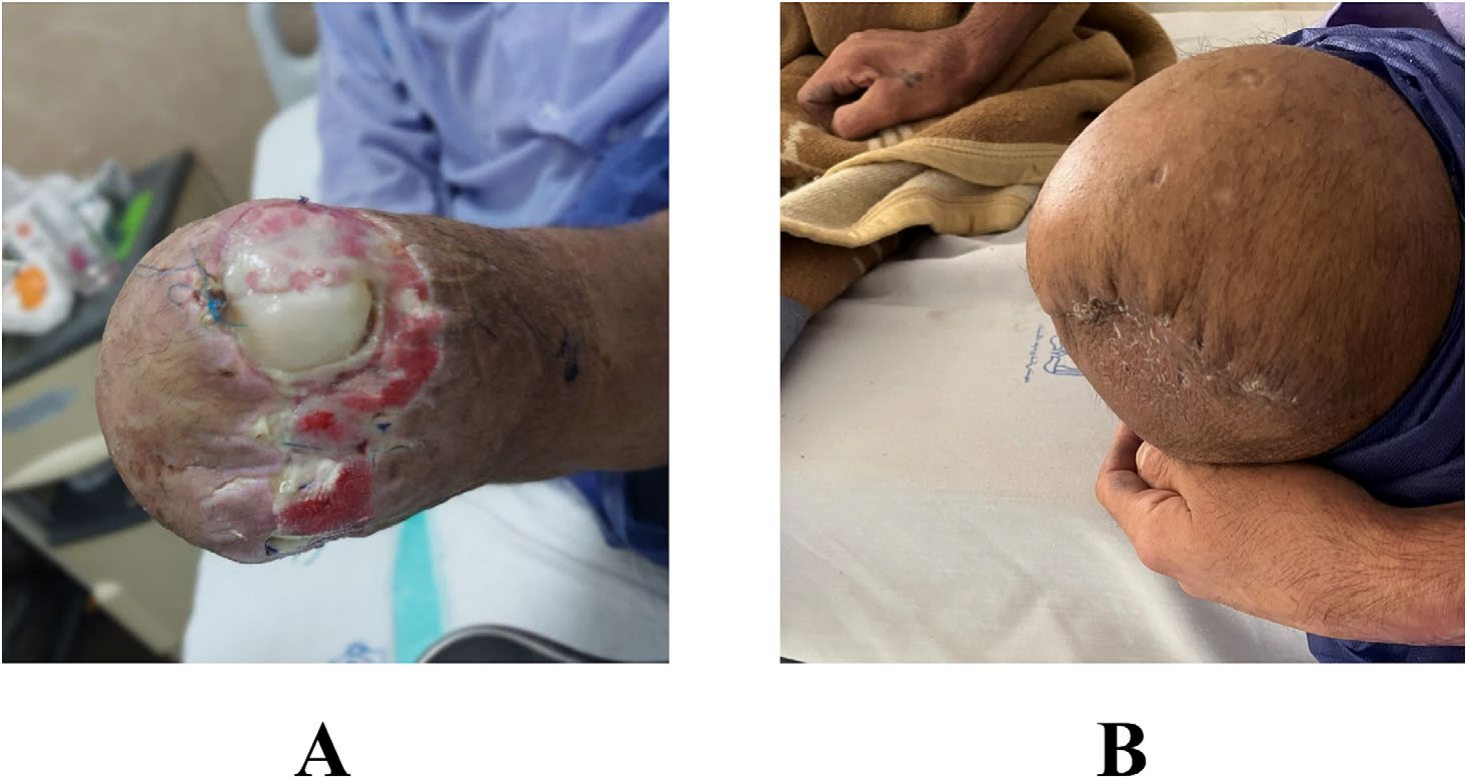
Wound images. (A) Patient's wound after the first amputation; (B) Complete treatment of the patient's wound.

After a complete evaluation and magnetic resonance imaging (MRI), the patient was diagnosed with multifocal osteomyelitis with extensive destruction of the knee. He received the appropriate type and dose of antibiotics for osteomyelitis (vancomycin 15 mg/kg and meropenem 1 g three times daily). The wounds were debrided, washed, and bandaged daily several times, but there was no improvement. After an orthopedic consultation, the patient's right leg was amputated at the above‐knee. After the amputation, the wounds again had purulent discharge (Figure 1A), so a second amputation was done from the middle of the femoral bone. Unfortunately, there was still purulent discharge from the amputation scars.

**FIGURE 2:**
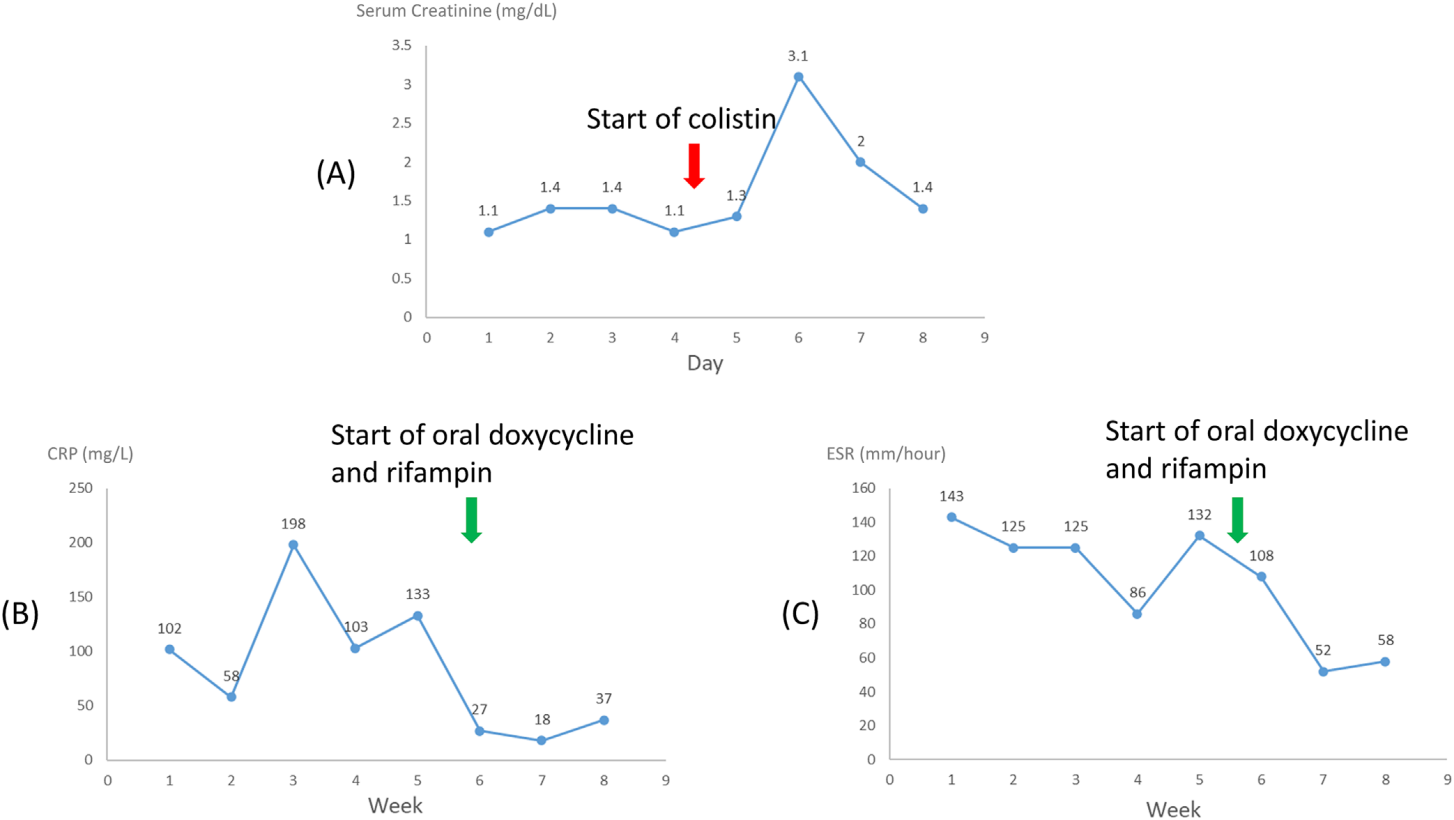
Laboratory trends. (A) Serum creatinine levels; (B) CRP levels; (C) ESR levels.

The culture of the bedside wound secretion swab was collected after 6 days of the second amputation. The isolate was identified as PDR *K. pneumoniae* . Antimicrobial susceptibility testing was performed using the routine CLSI‐based panel (including imipenem, meropenem, ertapenem, colistin, amikacin, gentamicin, ciprofloxacin, levofloxacin, trimethoprim‐sulfamethoxazole, cefepime, ceftazidime, piperacillin‐tazobactam, and tigecycline). The isolate was resistant to all tested agents. Colistin 9 million stat and 4.5 million twice daily along with extended‐infusion meropenem 1 g three times daily were started for the patient. A few days later, the patient's serum creatinine level started to increase (Figure 2A) and we had to hold the colistin as a result of acute kidney injury (AKI). At this time, a combination of oral doxycycline 100 mg twice daily and rifampin 600 mg once daily were started for the patient.

## 4 Conclusions and Results

Surprisingly, purulent discharges were improved completely a few days later (Figure 1B). ESR and CRP also declined (Figure 2B,C), which demonstrates the dramatic response of PDR *K. pneumoniae* to the combination of oral doxycycline and rifampin.

## 5 Discussion

In the present case, wound secretions culture showed the presence of PDR *K. pneumoniae* , a gram‐negative bacillus belonging to the Enterobacteriaceae, which was not sensitive to any antibiotic. According to the Infectious Diseases Society of America (IDSA) 2024 recommendation, meropenem‐vaborbactam, ceftazidime‐avibactam, imipenem‐cilastatin‐relebactam, or cefiderocol might be used for *K. pneumoniae* carbapenemases‐producing infection. Ceftazidime‐avibactam in combination with aztreonam, or cefiderocol alone, also might be utilized for metallo‐β‐lactamases‐producing Enterobacterales infections [2]. However, none of the mentioned antibiotics were available in our country. Genomic analysis for specific carbapenemase genes was not performed due to the lack of routine molecular diagnostic facilities in our center, which is a limitation of this report.

Tetracycline derivatives exhibit a broad antibacterial spectrum, including Gram‐negative bacteria. Tigecycline and eravacycline are considered alternatives to β‐lactam agents for the treatment of infections caused by carbapenem‐resistant enterobacterales [2]. However, both are less available and expensive. Doxycycline is also a well‐known and frequently used tetracycline derivative. A systematic review revealed promising effects of doxycycline from eight retrospective clinical studies in treating multidrug resistant (MDR) infections with Gram‐negative bacteria [3]. Thus, it might be used as an alternative for treating resistant Enterobacteriaceae. Rifampin, a bacterial RNA polymerase inhibitor antibiotic, is usually used for treating tuberculosis, brucellosis, and leprosy. Rifampin has demonstrated synergistic effects with multiple drugs in treating resistant gram‐negative bacterial infections [4]. In vitro, combinations of colistin and rifampin have demonstrated bactericidal and bacteriostatic properties against all strains of New Delhi metallo‐β‐lactamases‐1‐producing *K. pneumoniae* [5]. The combination of oral doxycycline and rifampin was utilized for our patient, which dramatically affected the clearance of PDR *K. pneumoniae* . Further clinical studies are needed to confirm this observation.

## Author Contributions

**Ali Akbar Heydari:** investigation, supervision, writing – review and editing. **Sepideh Elyasi:** supervision, writing – review and editing. **Farshad Abedi:** data curation, investigation, writing – original draft, writing – review and editing.

## Funding

The authors have nothing to report.

## Ethics Statement

This case report was conducted following the Declaration of Helsinki. Ethical approval was obtained from the Ethics Committee of ImamReza Hospital, Mashhad University of Medical Science (MUMS), Mashhad, Iran.

## Consent

The patient provided written informed consent for the publication of this case and any accompanying images.

## Conflicts of Interest

The authors declare no conflicts of interest.

## Data Availability

Please contact the correspondence for data requests.
